# The Scientific Treatment of Sea-Sickness

**Published:** 1912-07-20

**Authors:** 


### PRACTICAL POINTS.

(Criticism and Suggestions Invited.)

### The Scientific Treatment of Sea-sickness.

"Sea-sickness," wrote Charles Darwin, himself a martyr to the disease, "is no trifling evil to be cured in a week." This statement is not, perhaps, literally accurate, for there are varieties and types of sea-sickness, as every ship's doctor knows. Yet those who are liable to the complaint are justified in regarding it as a very serious matter, and the medical attendant who is called upon to treat them would do well to take an equally serious view of the case. Nothing is gained by making light of the patients' sufferings, and very often much mischief is done by dealing with all cases of sea-sickness in a routine indiscriminate way. If Boerhave's axiom—that "where many remedies are prescribed for a particular sickness be sure that no single remedy is of much use in curing that condition"—is true, then sea-sickness is a complaint for which modern science has at present no cure. But this statement, too, like Darwin's, needs modification. There are cases of sea-sickness which are easily cured by one or two home remedies, or even by simple mechanical expedients; there are others which resist all lines of treatment and seem to mock the skill of the doctor.

### The Worse Types.

Anyone who has had the misfortune to be called upon to treat one of these latter cases will readily agree that few clinical pictures are more harrowing than that presented by such cases. The writer remembers the case of a young woman, a passenger on one of the large mail boats from the East, whose sufferings commenced from the moment the vessel left port to the time when the first home harbour, Marseilles, was reached. This lady's condition was at times so critical that extraordinary precautions had to be taken to prevent her from succumbing to exhaustion, inanition, and shock. Saline infusions, galvanism, and injections of camphor and adrenalin were successful, but the experience was so awful that the patient decided to remain in Europe and not to return to the East. Such severe cases of sea-sickness present certain features which seem to show that the ordinary explanation of the disease—the theory of alterations in the nervous mechanism of the semicircular canals, leading to disturbance in the cerebellum—is not adequate to explain the etiology. That a nervous causation is probable in a large number of cases seems indisputable, but how the disturbance is brought about is still a matter of pure conjecture.

### Cure-alls for Sea-sickness.

A large number of specifics and nostrums are vaunted as infallible cures for sea-sickness. Every passenger has his or her own favourite remedy, and ships' officers, especially the doctor, are invariably asked for an opinion on one or other much advertised cure or preventive. While there can be no doubt that a few advertised specifics are very useful in many cases, their employment should be discouraged, since they nearly all contain powerful sedatives which are also cardiac depressants, and their use, except under medical supervision, is open to grave objection. Simple home measures, such as stuffing the ears with cotton-wool, drinking peppermint julep, a strong cup of hot coffee, or a stimulant before embarking, are useful in mild cases, but utterly fail in the more severe forms. A person who is liable to sea-sickness should be advised to take ordinary precautions before sailing. Not to overload the stomach with food or drink, to abstain from indigestible food or much alcohol, and to avoid fatigue, are essential preliminaries of the prophylactic treatment. Individual idiosyncrasy, as is well known, plays a prominent *rôle* in the etiology of the disease, and the ship's doctor should therefore consider and treat each case individually. It is useful to remember that vegetarians appear to suffer more severely from the disease than those whose diet is mixed: in their case a cup of strong beef-extract or a scraped meat sandwich before sailing is often very useful.

### Treatment in Mild Cases.

Rest—with the head lowered—in a comfortable position, with care that the extremities are kept warm, is imperative. It is sometimes necessary to give a full dose of a sedative or even a hypnotic to secure such undisturbed mental and physical rest. In mild cases a mixture of bromide of sodium and potassium, gr. x. aa., in water flavoured with compound tincture of cardamoms, is very effective. The addition of five minims of tincture of nux vomica to this mixture acts beneficially in some cases: in others the same quantity of spirits of nitrous ether is better. Chloral, sulphonal, adalin, veronal, and similar synthetic compounds are useful. When a more prolonged rest is required their use is always inadvisable, since they are all powerful drugs which are also powerful depressants. Where they are employed it is for obvious reasons advisable to disguise their presence or to keep the patient in ignorance of what he is taking. The writer has found the direct opium derivatives far more valuable in prophylaxis. Tincture of opium, morphia, or, best of all, omnopon, should be given a trial. An excellent preventive in many cases is three minims of omnopon taken on a lump of sugar half an hour before sailing, the dose being repeated twenty minutes later. This usually secures quiet and undisturbed repose, in some cases restful sleep, for five or six hours without the inconveniences of a morphia injection. It is a remedy that should, of course, only be used by the doctor.

### Severe Attacks.

In severe attacks of *mal de mer*, where profound exhaustion, with hæmatemesis and heart weakness, is the result of vigorous retching, omnopon is particularly indicated. The drug has a slightly bitter taste, but is well tolerated in most cases, and particularly so by children. In a case of a child of ten who suffered very severely from sea-sickness, and on whom bromides and the valerianates had no effect, omnopon in two-minim doses given as above directed acted with the best results.

Among the most useful adjuncts after the opium compounds are the valerianates. The popular validol, which is mainly composed of a valerian salt, is exceedingly useful; its action is much enhanced, apparently, when it is given in combination with strychnine. Almost equally efficacious is the B.P. tincture in drachm doses, with five minims of liquor strychninæ hydrochloridi added; one dose is usually insufficient, and the mixture will have to be repeated twice or thrice, with half-an-hour interval between the doses. Gerard's formula,* which succeeds where the valerianates fail in many cases, proves singularly useful; it is best given as a hypodermic injection, but can also be given in pill form. Hyoscyamus and hyoscine do not appear to have the slightest effect on severe cases; in mild cases they are hardly required. Paraldehyde, given in port wine, or better, in hot water flavoured with peppermint, sometimes proves of service, but most patients object very strongly to it.

### The Range of Remedies.

The choice of remedies in obstinate cases is very wide, but it is highly problematical if more than one or two drugs are really of any service in severe forms of the disease. The first essential is to keep up the patient's strength and to prevent exhaustion. Nutrient enemata are not of much use; if an enema is deemed necessary a glucose solution probably nourishes the patient as much as does an egg-and-milk injection. Strong freshly expressed beef-juice in teaspoon doses is perhaps the best food. Albumen water, with some good brandy added, is sometimes tolerated. Ice, both to suck and for epigastric compresses, is valuable, but more because it eases the patient than because it has any curative effect. Counter irritation is quite useless in the majority of cases. Where the vomiting is very troublesome, the best, and so far as the writer's experience goes the only, drug that can be relied upon is tincture of iodine in one-minim doses. This sometimes acts rapidly, but more often the retching persists and exhausts the patient to such an extent that cardiac stimulants have to be freely administered. In such extreme cases, saline infusions (not intravenous injections) are very useful; the addition of adrenalin to the saline fluid is to be recommended in cases where the pulse is weak and thready. The extreme sleeplessness in such cases is best combated by morphia or omnopon injections. Gastric lavage is useless cruelty in such cases. Cannabis indica, which has been lauded for its efficacy in some of these distressingly bad cases, can never be depended on; in a few cases it appears to act very efficaciously, while in others it is not of the slightest effect. Care should be taken that the patients are kept warm.

It is important to insist on proper tonic treatment when the patients are convalescent. The usual invigorating effects of sea air and the voyage may be depended on in most cases, but they should be supplemented by a course of iron and arsenic, good food and proper rest. During convalescence on a long trip many patients develop tonsillitis, which is best treated by the application of a mild solution of silver nitrate, and the use of chlorate of potash gargle subsequently.

* ℞ Strychnine, 0.001 gramme; atropine, 0.005 gramme.

